# Randomized Controlled Trials Evaluating Artificial Intelligence in Cardiovascular Care: A Systematic Review

**DOI:** 10.1016/j.jacadv.2025.102152

**Published:** 2025-09-24

**Authors:** Dor Hadida Barzilai, Karin Sudri, Gal Goshen, Eyal Klang, Eyal Zimlichman, Israel Barbash, Michal Cohen Shelly

**Affiliations:** a ARC Innovation Center, Sagol AI Hub, Sheba Medical Center, Ramat Gan, Israel; b Division of Data-Driven and Digital Medicine (D3M), Icahn School of Medicine at Mount Sinai, New York, New York, USA; c The Sheba Talpiot Medical Leadership Program, Sheba Medical Center, Ramat Gan, Israel; d Tel Aviv University, Faculty of Medicine, Tel Aviv, Israel; e Leviev Heart Center, Sheba Medical Center, Ramat Gan, Israel

**Keywords:** artificial intelligence, cardiovascular care, clinical outcome, machine learning, randomized controlled trial, systematic review

## Abstract

**Background:**

Artificial intelligence (AI) has shown promise in transforming health care, particularly in cardiology. However, there is a lack of high-quality evidence demonstrating its impact on crucial clinical outcomes.

**Objectives:**

The purpose of this study was to synthesize existing evidence from randomized controlled trials (RCTs) on the application of AI in cardiology, evaluating its impact on key clinical outcomes.

**Methods:**

We conducted a systematic review following Preferred Reporting Items for Systematic Reviews and Meta-Analyses (PRISMA) guidelines, searching MEDLINE, Web of Science, and the Cochrane Library from inception to November 2024. We included RCTs evaluating machine learning models compared to traditional methods in cardiovascular care. Primary outcomes focused on patient-important metrics, while secondary outcomes covered time and resource savings.

**Results:**

Eleven RCTs met the inclusion criteria. Studies were conducted between 2021 and 2024, with 81.2% being multicenter trials. Five studies (45.5%) reported improvements in clinical events, 6 (54.5%) showed enhanced diagnostic accuracy and early detection, and 3 (27.3%) demonstrated improved resource utilization.

**Conclusions:**

This review highlights AI's potential to enhance cardiovascular care through improved early detection, diagnostic accuracy, and resource efficiency. However, the limited number of RCTs indicates a need for more high-quality studies to validate AI’s effectiveness across various clinical domains.

Artificial intelligence (AI) technologies promise to transform health care by providing tools that can assist clinicians in making more accurate and timely decisions, ultimately improving patient outcomes. This potential is particularly evident in cardiology,^1^^,^^2^ a specialty characterized by its complexity and the abundance of data available from various diagnostic tools.^1^, ^2^, ^3^ AI applications in cardiology range from detecting arrhythmias^4^^,^^5^ and predicting heart failure to assessing the severity of coronary artery disease (CAD),^6^ imaging analysis, personalized treatment planning, and risk stratification. The primary advantage of AI lies in its ability to analyze vast amounts of data quickly and accurately, providing insights that may be beyond human capability.^7^

Despite the promising performance metrics of AI,^8^ there remains a lack of high-quality evidence demonstrating its impact on clinical outcomes such as mortality, hospitalization rates, major adverse cardiovascular events, treatment response rates, and patient-reported outcomes.^9^^,^^10^ The evidence gap is largely due to the limited number of prospective randomized controlled trials (RCTs) evaluating AI applications in real-world clinical settings.^11^ High-quality RCTs are essential to ensure that these technologies reliably improve patient outcomes in everyday practice.^9^^,^^11^, ^12^, ^13^ Several recent reviews have explored AI in cardiovascular care, but most included heterogeneous clinical domains, lacked systematic trial-level risk-of-bias assessment, or predated key reporting standards such as CONSORT-AI (Consolidated Standards of Reporting Trials–Artificial Intelligence) and SPIRIT-AI (Standard Protocol Items: Recommendations for Interventional Trials–Artificial Intelligence).^14^, ^15^, ^16^, ^17^, ^18^ To our knowledge, no prior review has focused exclusively on RCTs in cardiology while evaluating risk of bias and adherence to AI-specific reporting guidelines. This systematic review aims to synthesize the existing evidence from RCTs on the application of AI in cardiology, and specifically, to identify which AI interventions have been validated against gold standard practices, assess their impact on key clinical outcomes, and pinpoint gaps that should guide future research.

## Methods

### Search strategy

We conducted a systematic review following the Preferred Reporting Items for Systematic Reviews and Meta-Analyses (PRISMA) guidelines for critical appraisal and data extraction.^19^ We searched peer-reviewed original articles evaluating the use of machine learning models compared to traditional methods in various applications of cardiovascular care as evaluated by RCTs. The search was performed using MEDLINE, Web of Science, and the Cochrane Library from inception to November 2024, using keywords such as “Artificial intelligence,” “Myocardial infarction,” “Heart failure,” and “Randomized controlled trial,” Supplemental Appendix 1 provides the complete search strategy including all keywords used. Supplemental Appendix 2 represents the PRISMA checklist. The data underlying this systematic review are derived from publicly available sources and do not require Institutional Review Board approval.

### Study selection and data extraction

**Figure 1:**
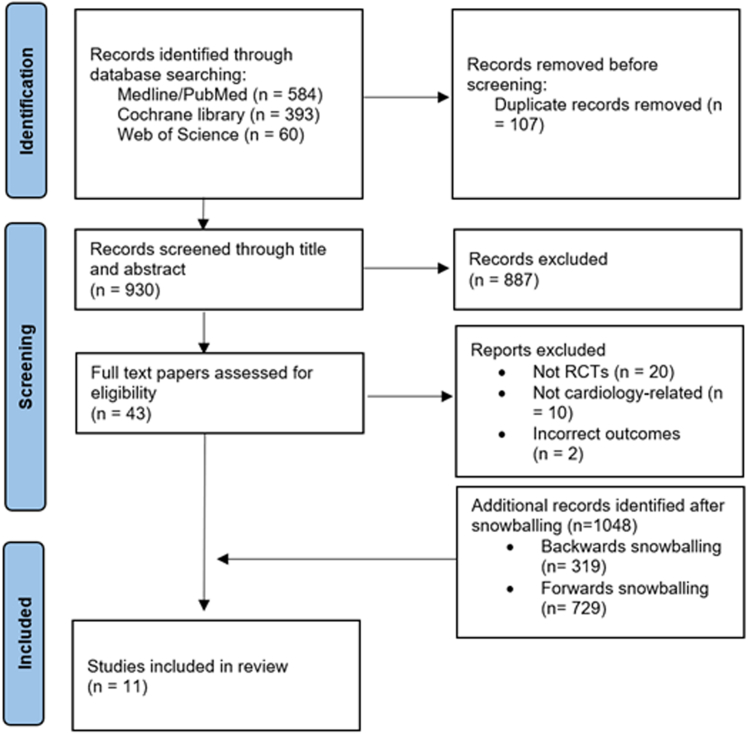
**PRISMA Flow Diagram** Studies were excluded based on title and abstract screening focusing on RCTs that use machine learning in cardiology. PRISMA = Preferred Reporting Items for Systematic Reviews and Meta-Analyses; RCTs = randomized controlled trials.

Two authors independently reviewed titles and abstracts based on predetermined eligibility criteria documented in our study protocol registered with PROSPERO (International Prospective Register of Systematic Reviews) (CRD42024548371). We included only peer-reviewed, full-text articles of RCTs that evaluate the use of machine learning models in cardiovascular care. A third reviewer resolved disagreements between the 2 reviewers. Backward and forward snowballing techniques were employed, examining reference lists and citation tracking of the included articles to identify additional relevant studies. The 2 authors then independently evaluated the full-text studies. To avoid duplication and ensure data integrity, we screened for overlapping populations and multiple publications by cross-referencing trial registration numbers, study settings, sample sizes, recruitment periods, and author lists. No duplicate reports were identified, and each included RCT represented a unique study. Figure 1 illustrates a flowchart of the screening and inclusion process. We utilized a standardized data extraction sheet to gather relevant information from the reviewed studies (Table 1).

**Table 1:** Metadata of the Included Trials

First Author	Title	Journal	Country	Year of Publication
Yao et al^25^	Artificial intelligence-enabled electrocardiograms for identification of patients with low ejection fraction: a pragmatic, randomized clinical trial	*Nature Medicine*	USA	2021
Huang et al^29^	Portable Device Improves the Detection of Atrial Fibrillation After Ablation	*International Heart Journal*	China	2021
Hill et al^26^	Identification of undiagnosed atrial fibrillation using a machine learning risk prediction algorithm and diagnostic testing (pulse-AI) in primary care: a multi-centre randomized controlled trial in England	*European Heart Journal - Digital Health*	England	2022
Sandhu et al^31^	Incidental Coronary Artery Calcium: Opportunistic Screening of Previous Nongated Chest Computed Tomography Scans to Improve Statin Rates (NOTIFY-1 Project)	*Circulation*	USA	2022
De Backer et al^32^	Impact of Computational Modeling on Transcatheter Left Atrial Appendage Closure Efficiency and Outcomes	*JACC cardiovascular interventions*	Denmark	2023
He et al^33^	Blinded, randomized trial of sonographer vs AI cardiac function assessment	*Nature Medicine*	USA	2023
Yang et al^30^	On-Site Computed Tomography–Derived Fractional Flow Reserve to Guide Management Of patients with Stable Coronary Artery Disease: The TARGET Randomized Trial	*Circulation*	China	2023
Lin et al (2024a)^24^	AI-enabled electrocardiography alert Intervention and all-cause mortality: a pragmatic randomized clinical trial	*Nature Medicine*	Taiwan	2024
Adedinsewo et al^27^	Artificial intelligence-guided screening for Cardiomyopathies in an obstetric population: a pragmatic randomized clinical trial	*Nature Medicine*	Nigeria	2024
Lin et al (2024b)^28^	Artificial Intelligence-Powered Rapid ST-Elevation Myocardial Infarction Identification via Electrocardiogram (ARISE): A Pragmatic Randomized Controlled Trial	*NEJM AI*	Taiwan	2024
Upton et al^34^	PROTEUS: A Prospective RCT Evaluating Use of AI in Stress Echocardiography	*NEJM AI*	England	2024

### Outcomes and evaluation metrics

Primary outcomes focused on patient-important metrics, including mortality, hospitalization rates, major adverse cardiovascular events, treatment adherence, and early diagnosis, as RCTs are considered the gold standard for evaluating improvements in clinical management.^16^ Secondary outcomes addressed the optimization of resource allocation, such as time and cost savings and reductions in manpower, reflecting the transformative role of AI in reshaping health care operations.^20^ Notably, none of the included studies assessed patient-reported outcome measures, which represent a critical dimension in evaluating the effectiveness of health care interventions.^21^

### Risk of bias

The Quality Assessment of Diagnostic Accuracy Studies-2 (QUADAS-2) tool was utilized to systematically evaluate the quality and diagnostic accuracy of the studies under review across 4 critical domains: patient selection, index test, reference standard, and flow and timing. In addition, we also assessed the risk of bias related to funding and conflicts of interest. Although QUADAS-AI remains under development, it is expected to serve as a vital tool for evaluating the risk of bias in AI-driven diagnostic accuracy studies.^22^^,^^23^

The quality of randomization varied across studies, from simple methods (eg, random number tables, record-based assignment) to more robust techniques like stratified or block randomization with independent oversight. This heterogeneity may influence the internal validity of findings and highlights the need for transparent reporting in future AI-related RCTs. A summary of randomization methods is provided in Supplemental Table 3.

## Results

**Figure 2:**
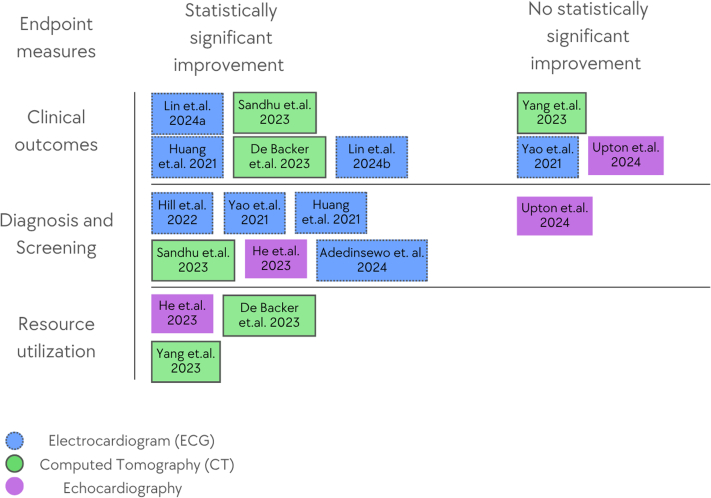
Summary of Included Studies by Endpoint and Result The matrix groups studies by endpoint category, clinical outcomes, diagnosis and screening, or resource utilization, and by whether the artificial intelligence intervention showed a statistically significant improvement or no statistically significant improvement. Each tile represents one study labeled by first author and year.

Our search retrieved 930 study records after deduplication (Figure 1). After screening titles and abstracts, 43 articles were retained for full-text review. Of these, 32 were excluded, leaving 11 RCTs included in our systematic review. The geographic distribution of these studies was 3 from the United States (37.5%), 2 from China, England, and Taiwan (each 18.2%), and 1 each from Nigeria and Denmark (12.5%). The studies were conducted between 2021 and 2024. Most studies (81.2%) were multicenter trials. The most common AI application was to electrocardiography (ECG) data (54.5%), followed by computed tomography (27.3%) and echocardiography (18.2%). Studies were distributed between diagnosis, prevention, and screening (72.7%) and procedural optimization and resource efficiency (27.3%) (Table 2, Figure 2).

**Table 2:** Technical Information About the Included Trials

First Author	Main Domain	Study Design	Single/Multisite	Number of Participants	AI/Prediction Task	Study Objective	Added Value With AI	Ethical Statement	Funding	Conflict of Interest
Yao et al^25^	ECG, prevention, and screening	Prospective RCT	Multisite	358 clinicians and 22,641 patients	AI-ECG alert for early diagnosis of low LVEF	To evaluate whether an AI-enabled ECG can serve as a screening tool detecting low EF in routine practice.	Increased the diagnosis of low EF (1.6% in the control arm vs 2.1% in the intervention arm). In patients with a high likelihood of low EF (14.5% vs 19.5%, respectively)	IRB approval and informed consent were obtained	The Mayo Clinic Robert D. And Patricia E. Kern Center for the Science of Health Care Delivery	Multiple authors declare financial benefits from using the AI-ECG system.
Huang et al^29^	ECG, prevention and screening	Prospective RCT	Single center	218 patients	AI-enabled automated AF detection	Detection of asymptomatic recurrences of AF after ablation by a handheld, AI-enabled, ECG monitor	AF-free survival rates were 64.2% in the test group and 78.9% in the control group (*P* = 0.0163). There was greater adherence to oral anticoagulation in the test group (51.0% vs 25.4%, *P* = 0.0052).	IRB approval and informed consent were obtained	The National Natural Science Foundation of China	None
Hill et al^26^	ECG, prevention, and screening	Prospective RCT	Multisite	23,745 patients	Machine learning risk prediction for undiagnosed AF	To evaluate a machine learning algorithm for detecting AF in a primary care setting.	9.41% of the intervention group received AF and related arrhythmia diagnoses vs 4.93% in the control group	IRB approval and informed consent were obtained	Bristol Myers Squibb Pharmaceuticals Ltd and Pfizer. One of the authors is funded by the NIHR Biomedical Research, Centre, Oxford.	Multiple authors declared a conflict of interest.
Sandhu et al^31^	CT, prevention and screening	Prospective RCT	Multisite	2,113 patients	AI-based opportunistic screening for coronary artery calcium on a nongated chest CT	To evaluate the effect of notifying clinicians about high-risk patients with incidental coronary artery calcium	Statins were prescribed to 51.2% of the notification arm vs 6.9% of the usual care arm (*P* < 0.001). 15.1% of the notification arm underwent testing for CAD compared to 2.3% in the usual care arm (*P* = 0.008).	Exempt from human subject research requirements by the Stanford IRB.	The Stanford University Human-Centered Artificial Intelligence Seed Grant.	Multiple authors declared a conflict of interest.
De Backer et al^32^	CT, cardiac device implantation	Prospective RCT	Multisite	200 patients	AI-enabled CT-based computational modeling for planning transcatheter left atrial appendage closure	To assess the effect of AI-driven CT-based computational modeling in planning transcatheter left atrial appendage closure on procedural efficiency and patient outcomes.	**Improved patient outcome:** complete left atrial appendage closure with no residual leak or disc retraction was observed in 44.0% of cases vs 61.1% (RR: 1.44; 95% CI: 1.05-1.98; *P* = 0.03). **Improved procedural efficiency**: fewer Amulet devices used (103 vs 118; *P* < 0.001) and fewer device repositioning (104 vs 195; *P* < 0.001) in the CT + simulation group.	IRB approval	Abbott (United States) and Feops NV (Belgium)	Multiple authors declared a conflict of interest
He et al^33^	Echocardiography, diagnosis	Retrospective RCT	Single center	25 sonographers, 10 cardiologists, and 3,495 patients	AI initial interpretation workflow of echocardiography and LVEF	To compare the accuracy and efficiency of initial LVEF assessments by AI vs sonographers.	The cardiologist's final assessment significantly altered 16.8% of AI group studies and 27.2% of the sonographer’s group (95% CI: −13.2% to −7.7%). The AI-guided workflow reduced the time required for both sonographers and cardiologists.	IRB approval with waiver of individual consent	No external funding was obtained for this study	Stanford University is in the process of applying for a patent application covering video-based deep learning models for assessing cardiac function
Yang et al^30^	CT-fractional flow reserve, diagnosis	Prospective RCT	Multisite	1,216 patients	Identification of CAD in CT-fractional flow reserve	To compare the on-site CT-fractional flow reserve strategy using machine learning to standard care (stress test) in diagnosing stable CAD.	The intervention group had a lower percentage of patients undergoing ICA without obstructive disease (28.3% vs 46.2%; *P* < 0.001).	IRB approval and informed consent were obtained	The National Key Research and Development Program of China and the Beijing Nova Program	None
Lin et al (2024a)^24^	ECG, prevention, and screening	Prospective RCT	Multisite	39 clinicians and 15,965 patients	AI-enabled ECG to identify hospitalized patients with a high risk of mortality	To evaluate whether an AI-enabled ECG alert can reduce all-cause mortality among high-risk hospitalized patients	All-cause mortality reduction - 3.6% in the intervention group vs 4.3% in the control group. For high-risk patients, there is a reduction in the risk of cardiac death (0.2% vs 2.4%, respectively)	IRB approval and informed consent were obtained	The National Science and Technology Council, Taiwan; The Cheng Hsin General Hospital, Taiwan; and the Medical Affairs Bureau, Taiwan.	Multiple authors declare financial benefits from using the AI-ECG system.
Adedinsewo et al^27^	Auscultation and ECG, prevention and screening	Prospective RCT	Multisite	1,232 patients	AI-enabled digital stethoscope and ECG to identify LV systolic dysfunction	To assess AI-guided screening for peripartum cardiomyopathy compared to standard of care	LV systolic dysfunction was detected in 4.1% of the intervention group vs 2.0% of the control group (*P* = 0.032)	IRB approval and informed consent were obtained	The Mayo Clinic and the National Institutes of Health	Multiple authors declare financial benefits from using the AI-ECG system
Lin et al (2024b)^28^	ECG	Prospective RCT	Multisite	43,234 patients	AI-ECG to detect STEMI	To evaluate the impact of AI-ECG-guided alerts on treatment delay and diagnostic accuracy in STEMI cases	For patients in the emergency department, the median door-to-balloon time was 82.0 minutes in the intervention group compared with 96.0 minutes in the control group (*P* = 0.002).	IRB approval, with informed consent waived	The National Science and Technology Council, Taiwan	None
Upton et al^34^	Echocardiography	Prospective RCT	Multisite	2,341 patients	AI-augmented interpretation of stress echocardiography	To evaluate the accuracy of AI-augmented interpretation of stress echocardiography compared to standard care	None	IRB approval and informed consent were obtained	Accelerated Access Collaborative, NHSX, and the National Institute for Health Research	Multiple authors declared a conflict of interest.

AF = atrial fibrillation; AI = artificial intelligence; CAD = coronary artery disease; ECG = electrocardiography; EF = ejection fraction; IRB = Institutional Review Board; LV = left ventricle; RCT = randomized controlled trial.

### Outcomes by imaging modality

Synthesizing outcomes across imaging modalities revealed distinct patterns of AI effectiveness in cardiovascular applications.

ECG-based AI applications (6 studies, 54.5%) demonstrated consistent clinical benefits across diverse contexts: mortality reduction in hospitalized patients (HR: 0.83),^24^ improved detection of low ejection fraction (EF) (OR: 1.32),^25^ enhanced arrhythmia diagnosis (OR: 2.24),^26^ increased left ventricle (LV) dysfunction detection in peripartum women (OR: 2.12),^27^ and reduced door-to-balloon times in ST-segment elevation myocardial infarction (STEMI) (14.6% reduction),^28^ though with mixed results for postablation monitoring.^29^

Computed tomography (CT)-based AI applications (3 studies, 27.3%) showed strongest impact on clinical workflows and resource optimization, significantly reducing unnecessary invasive coronary angiography (39.4% reduction),^30^ increasing preventive therapy adoption (7.4-fold increase in statin prescriptions),^31^ and improving procedural outcomes in device implantation (38.9% improvement in success rate) while reducing device use and manipulation.^32^

Echocardiography AI applications (2 studies, 18.2%) demonstrated contrasting outcomes: significant workflow efficiency gains and reduced interpretation variability in routine assessments (38.2% fewer substantial changes between initial and final assessments),^33^ but insufficient diagnostic performance in stress echocardiography for CAD detection.^34^

### Clinical outcomes versus operational benefit

AI interventions frequently produced stronger operational than clinical outcomes. Operationally, AI consistently enhanced workflow efficiency by reducing variability in echocardiographic interpretation,^33^ shortening door-to-balloon time in STEMI,^28^ and optimizing procedural resource use in CT-guided device implantation.^32^ In contrast, improvements in clinical endpoints, such as reduced mortality,^24^ were less frequent, often modest in magnitude, or demonstrated significance primarily in subgroup analyses.

### AI in population-based settings versus specialized clinical contexts

AI's effectiveness varied by clinical setting. Studies conducted in high-volume settings, such as primary care atrial fibrillation (AF) screening,^26^ population-based low EF detection,^25^ and preventive coronary artery calcium (CAC) screening,^31^ demonstrated consistent diagnostic and preventive therapy benefits, highlighting AI’s strength in managing standardized, large-scale data. Conversely, specialized procedural contexts, such as stress echocardiography for CAD,^34^ showed more limited diagnostic accuracy, indicating complexities that may require more tailored AI algorithms or integrated human oversight.

### Early detection and prevention

Across seven RCTs examining early detection and prevention, AI interventions consistently improved diagnostic rates and accelerated identification of high-risk patients, especially in large-scale screening contexts. ECG-based algorithms notably enhanced detection rates of clinically significant cardiac conditions such as low EF, AF, and myocardial infarction, facilitating timely interventions. While absolute improvements in some outcomes (eg, mortality) were modest, AI tools demonstrated clear value in risk stratification, significantly increasing targeted downstream testing and therapy initiation. These consistent, directional benefits across varied clinical settings indicate AI’s potential as an effective frontline screening and preventive tool.

Hill et al^26^ conducted a study involving 23,745 participants across 6 primary care clinics in England, where machine learning–based AF screening in the intervention arm identified high-risk patients for diagnostic testing. AF and related arrhythmias were diagnosed in 5.63% of high-risk participants in the intervention group, compared to 4.93% in the control arm (OR: 1.15; 95% CI: 0.77-1.73; *P* = 0.486). High-risk participants in the intervention arm who underwent diagnostic testing were twice as likely to receive arrhythmia diagnoses compared to routine care (9.41% vs 4.93%; OR: 2.24; 95% CI: 1.31-3.73; *P* = 0.003).

Another RCT by Yao et al^25^ with 22,641 patients from 45 Mayo Clinic practices assigned the intervention group AI alerts for low EF based on ECGs, while the control group received standard care. The intervention group had a higher diagnosis rate of low EF (2.1%) compared to the control group (1.6%) (OR: 1.32; 95% CI: 1.08-1.61; *P* = 0.007). Echocardiography utilization was higher in the intervention group (49.6% vs 38.1%; *P* < 0.001), as was the diagnosis rate of low EF (19.5% vs 14.5%; OR: 1.43; 95% CI: 1.08-1.91; *P* = 0.01). Similarly, Lin et al (2024a)^24^ conducted a multisite RCT involving 15,965 patients at both an academic and a community hospital in Taiwan, with the intervention group receiving AI-ECG alerts for high mortality risk, prompting intensive care, while the control group received standard care. The intervention group showed a significant reduction in all-cause mortality within 90 days (3.6% vs 4.3%; HR: 0.83; 95% CI: 0.70-0.99; *P* = 0.04). Among high-risk patients, mortality fell from 23.0% to 16.0% (HR: 0.69; 95% CI: 0.53-0.90; *P* = 0.006). Increased Intensive Care Unit (ICU) admissions (HR: 1.40; 95% CI: 1.06-1.85; *P* = 0.016) and more frequent echocardiography (HR: 1.36; 95% CI: 1.15-1.61; *P* < 0.001) were observed.

Huang et al^29^ evaluated the detection of asymptomatic AF recurrences using a handheld AI-enabled ECG monitor (BigThumb) in postablation patients. 218 participants were randomized into an intervention arm using BigThumb and a control arm with traditional follow-up. The AF-free survival rate was higher in the control arm (78.9% vs 64.2%; *P* = 0.0163). Adherence to oral anticoagulation was better in the intervention arm (51.0% vs 25.4%; *P* = 0.0052).

Sandhu et al^31^ implemented a deep learning algorithm for CAC screening in patients without known atherosclerotic cardiovascular disease who had a nongated chest CT scan. Participants with positive CAC were randomized into notification (intervention) or usual care (control) arms. The intervention significantly increased statin prescriptions (51.2% vs 6.9%; *P* < 0.001) and CAD testing (15.1% vs 2.3%; *P* = 0.008), with lower low-density lipoprotein (LDL) levels during follow-up in the intervention group (97.2 mg/dL [SD: 30.3] vs 115.3 mg/dL [SD: 29.4], *P* = 0.005, respectively).

Lin et al (2024b)^28^ conducted another multicenter RCT involving 43,234 patients across 2 medical centers in Taiwan to evaluate AI-ECG for STEMI identification and alert, focusing on treatment time reduction. Patients were cluster-randomized daily, with on-duty cardiologists in the intervention group receiving SMS alerts for potential STEMI cases identified by the system, while the control group followed standard care. The intervention significantly reduced door-to-balloon time from 96.0 to 82.0 minutes (*P* = 0.002). Although no significant differences were observed in secondary clinical outcomes, the AI-ECG system demonstrated a high positive predictive value of 89.5% and a negative predictive value of 99.9%.

Adedinsewo et al^27^ conducted a pragmatic, RCT in 6 hospitals in Nigeria to assess Food and Drug Administration (FDA) - approved, AI-guided screening for peripartum cardiomyopathy in 1,232 women, comparing AI ECG and stethoscope screening to standard care. AI-guided screening detected LV systolic dysfunction in 4.1% of women in the intervention group vs 2.0% in the control group (OR: 2.12; 95% CI: 1.05-4.27; *P* = 0.032). Although the AI-ECG alone did not show a statistically significant difference, the digital stethoscope demonstrated 95.7% sensitivity and Area Under the Curve (AUC) of 0.976. The intervention required a number needed-to-screen of 47 to detect one additional LV systolic dysfunction case, highlighting its utility in low-resource settings.

Upton et al^34^ performed a multicenter RCT at 20 centers across the United Kingdom involving 2,341 participants undergoing stress echocardiography for significant CAD. Participants were randomized into an intervention group, where AI was used to augment test interpretation, or a control group, where tests were interpreted per standard protocol. The AI-augmented approach showed an Area Under the Receiver Operating Characteristic (AUROC) of 0.63 (95% CI: 0.43-0.83) compared to 0.55 (95% CI, 0.33-0.80) in the control group, which did not meet the noninferiority margin for detecting acute coronary events.

### Procedural optimization and resource efficiency

Three RCTs demonstrated AI’s substantial role in optimizing procedural workflows and resource utilization. AI-driven echocardiography and CT-based planning significantly reduced procedural variability, shortened interpretation and procedure times, decreased the frequency of unnecessary invasive procedures, and improved device implantation outcomes. Although clinical outcomes such as major cardiovascular events were not significantly impacted, operational efficiency gains, including reduced clinician workload and procedural costs, show AI’s immediate practical benefits. These results suggest a meaningful role for AI as a complement to clinician expertise, enhancing efficiency, consistency, and resource management, especially in high-volume cardiovascular care settings.

He et al^33^ compared AI with sonographers for initial echocardiography assessments in a noninferiority trial with 3,495 studies, using the cardiologist’s final assessment as the reference. The AI group had a significantly lower proportion of substantial changes between initial and final left ventricular ejection fraction (LVEF) assessments (16.8% vs 27.2%; 95% CI: −13.2% to −7.7%; *P* < 0.001), with a lower absolute difference in LVEF (2.79% vs 3.77%; *P* < 0.001). Notable time savings were observed for sonographers (median 119s for AI vs 0s; *P* < 0.001) and cardiologists (median 54s for AI vs 64s, *P* < 0.001). The study demonstrates that AI-guided assessments are noninferior to sonographer assessments, with benefits in both accuracy and efficiency.

De Backer et al^32^ investigated the use of AI-enabled CT planning for transcatheter left atrial appendage closure. The study randomized 200 patients to standard planning or CT simulation-based planning. The AI group showed improved patient outcomes with a higher complete closure rate (61.1% vs 44.0%; relative risk (RR): 1.44; 95% CI: 1.05-1.98; *P* = 0.03). Procedural efficiency improved with 15% fewer devices used (103 vs 118; *P* < 0.001) and a 50% reduction in device repositioning (104 vs 195; *P* < 0.001).

Yang et al^30^ compared an on-site CT-fractional flow reserve strategy using machine learning vs a standard stress test in the diagnosis of stable CAD. The intervention group had fewer patients referred for angiography without obstructive CAD or intervention within 90 days (28.3% vs 46.2%; *P* < 0.001). Major adverse cardiovascular events at 1 year did not significantly differ (HR: 0.88; 95% CI: 0.59-1.30).

### Risk of bias

**Figure 3:**
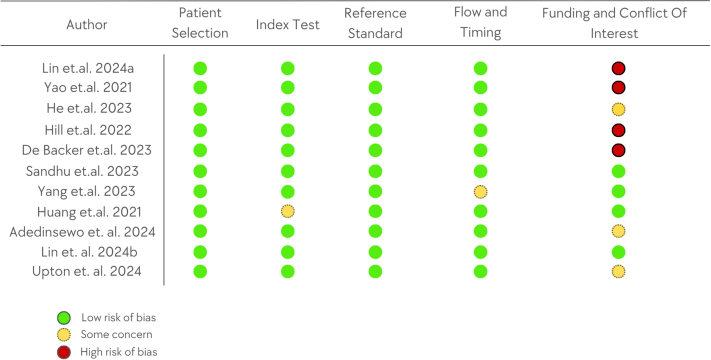
**Assessment of the Risk of Bias** The QUADAS-2 tool was used to evaluate the risk of bias in patient selection, the index test, the reference standard, and flow and timing. In addition, assessment of the risk of bias due to funding and conflict of interest. QUADAS-2 = The Quality Assessment of Diagnostic Accuracy Studies-2.

The QUADAS-2 tool was used to evaluate the risk of bias in patient selection, the index test, the reference standard, and flow and timing (Figure 3). Regarding patient selection, all the included studies are RCTs with well-defined population selection criteria that adhered to a randomization process, most of them conducted across multiple centers. In the evaluation of the index test, Huang et al used a portable device to detect AF after an ablation procedure. The device's monitoring frequency was lower during nighttime, which could indicate a potential inconsistency in how the index test (BigThumb ECG device) was applied. Other than this, all studies applied a consistent methodology for the index test by the utilization of AI. Regarding the reference test risk of bias, the eleven studies consistently used either the gold standard test or actual clinical outcomes as the reference test, indicating the lowest risk of bias. In terms of flow and timing, Yang et al used routine chest CT as a screening tool for CAC. The extended time gap (median of 857 days) between the initial chest CT scan and the notification of CAC findings could potentially impact the effectiveness of the intervention, as the timely initiation of preventive measures, such as statin therapy, is crucial for reducing cardiovascular risk. In addition, we assessed the risk of bias related to funding and conflicts of interest. In this regard, seven out of eleven studies (63.6%) were found to have a moderate to high risk of bias, indicated by potential personal gain for the authors or the funding organizations from the successful implementation of the AI tools. In addition, only 5 of 11 studies declared adherence to AI-specific reporting guidelines such as CONSORT-AI and SPIRIT-AI.

As AI in health care research matures, several reporting guidelines, including CONSORT-AI, SPIRIT-AI, PRISMA-AI, Standards for Reporting Diagnostic Accuracy — Artificial Intelligence (STARD-AI), and others have been introduced or are under development.^35^, ^36^, ^37^, ^38^, ^39^, ^40^, ^41^, ^42^, ^43^, ^44^ These tools aim to standardize and enhance the quality, transparency, and reproducibility of AI-related clinical research. Our review found that only 5 of 11 RCTs adhered to SPIRIT-AI or CONSORT-AI, demonstrating a gap in standardization. A summary of the different guidelines and adherence is in Supplemental Tables 4 and 5. This lack of alignment with established reporting underscores the urgent need for broader adoption of these standards in future studies.

## Discussion

Our systematic review evaluates the objective impact of AI in cardiology, focusing on the outcomes reported in RCTs. After a screening and full-text review process, eleven RCTs were included. These studies explored various AI applications across different modalities, such as ECG, echocardiography, and cardiac CT, while measuring a range of clinical and operational outcomes. Five studies (45.5%) reported improvements in clinical outcomes, 6 studies (54.5%) demonstrated enhanced diagnostic accuracy and early detection, and 3 studies (27.3%) showed improved resource utilization (Central Illustration).

**Central Illustration:**
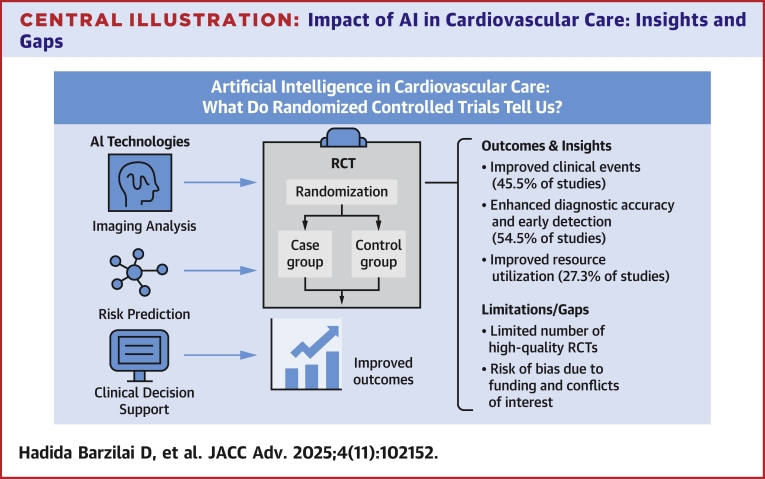
**Impact of AI in Cardiovascular Care: Insights and Gaps** This central illustration summarizes the key findings of our systematic review of randomized controlled trials evaluating AI applications in cardiovascular care. It highlights the main AI technologies (imaging analysis, risk prediction, and clinical decision support) and illustrates their flow into randomized trial designs. Outcomes and insights include improved clinical events (45.5% of studies), enhanced diagnostic accuracy and early detection (54.5%), and improved resource utilization (27.3%). Key limitations and gaps identified in the current evidence include the limited number of high-quality RCTs and potential bias due to funding and conflicts of interest. AI = artificial intelligence; other abbreviation as in Figure 1.

Previous systematic reviews have shown the added value of leveraging AI in clinical practice to improve prediction and diagnostic accuracy, as well as personalize treatment strategies in various modalities such as heart failure and cardiomyopathy detection,^45^ left ventricular hypertrophy identification,^46^ interpretation of ECG,^47^ and cardiac stress tests,^8^ valvular disease diagnosis,^48^ and transcatheter aortic valve implantation risk stratification.^49^ Despite significant research indicating that medical AI models can perform as well as or better than clinicians across various tasks and specialties, some of these models have only been tested retrospectively. In addition, several recent reviews have surveyed AI in cardiology: Moosavi et al^17^ offered a scoping review through 2023, Zhou et al^16^ systematically examined AI RCTs across health care before CONSORT-AI, Plana et al^15^ covered machine learning trials broadly through 2021 without emphasis on cardiovascular care, and Elias, Jain et al^14^^,^^18^ provided narrative advances without methodological appraisal. Yet none focus solely on cardiovascular RCTs published up to November 2024, apply a tailored QUADAS-2 risk-of-bias assessment including randomization audits and funding/conflict of interest analyses, or evaluate adherence to CONSORT-AI and SPIRIT-AI guidelines. Our review directly addresses these limitations, delivering the most current, comprehensive synthesis of randomized evidence for AI’s real-world clinical impact in cardiovascular care.

To integrate AI-based applications into routine clinical practice, their added value must be reliably demonstrated through rigorous evaluation. In particular, AI tools must show improvement in clinical outcomes such as reductions in mortality, hospitalization, and major adverse cardiovascular events or offer clear operational benefits, including enhanced efficiency, reduced clinician workload, or lower health care costs. These capabilities must be confirmed in prospective, well-designed randomized trials to ensure both efficacy and real-world applicability. As an alternative to clinical outcomes, these technologies should prove to be as good as a physician but with the ability to reduce costs or improve clinician time resources. This characteristic needs to be proven by clinical studies. Well-designed studies with robust methodologies are pivotal to accurately assess real-world clinical outcomes, which might differ when testing a machine learning model in lab settings. Hence, RCTs are crucial for validating those AI algorithms across diverse populations, data sources, and devices, ensuring generalizability and reducing bias.^50^ They also demonstrate the value proposition of AI tools in real-world settings, showing how AI can improve clinical workflows by saving time, reducing hospitalizations, and enabling early diagnosis and intervention.

A clear assessment of the risk of bias in these studies is crucial. As shown by Han et al, there is growing use of AI in clinical trials with promising results, but ensuring practical application in health care requires more than just broad external validation. Instead of focusing solely on multicenter studies, we must prioritize reliability across sites through recurring local validation, diverse outcome measures, and improved reporting to address the challenges of generalizability and site-specific performance.^11^^,^^51^ They demonstrated that a significant proportion of AI RCTs are concentrated in gastroenterology (43%) and radiology (13%), with most studies conducted in the United States, followed by China. There's also a notable gap in primary care research compared to specialty care, presenting an opportunity for future studies. Learning from other fields like gastroenterology and radiology, which have concentrated a significant number of AI RCTs, can guide better trial design in cardiology by incorporating lessons on trial structure, data diversity, and outcome validation.^52^ In this systematic review, we also highlight the risk of bias related to funding and conflicts of interest. To mitigate potential biases from industry involvement, hospitals and clinical key opinion leaders must take a proactive role in conducting and leading AI research. Their involvement can help maintain scientific rigor, ensure patient-centered outcomes, and reduce the influence of commercial interests. By fostering a culture of clinical leadership in AI research, health care institutions can become leaders in driving the responsible integration of AI into medical practice.

### Limitations of current evidence and future research directions

AI has the potential to transform clinical practice significantly; however, several challenges must be addressed to fully realize its benefits. A primary concern is the availability of high-quality medical data. Most RCTs have focused on imaging data or ECG pattern recognition rather than broader clinical data. This narrow focus may be due to difficulties in accessing large-scale, structured clinical data sets like electronic health records. The lack of comprehensive, high-quality clinical data limits the development of AI models that could incorporate a wider range of patient information, such as demographics and lab results. Future research should aim to utilize these data sources to create more personalized and actionable AI tools for patient care.

Issues related to data privacy and confidentiality, informed consent, and patient autonomy are particularly pressing. Additionally, the increasing threat of cybersecurity breaches requires caution and the reinforcement of protective measures. It is crucial to influence the development of ethical and legal guidelines to ensure the safe integration of deep learning technologies in medical practice. Addressing these challenges will be vital for fostering trust among patients and maintaining the integrity of the clinician-patient relationship.^9^^,^^53^

Our systematic review is constrained by several limitations. This is a rapidly evolving field, with all the studies identified published within the last 3 years, reflecting the novelty of AI applications in cardiology. However, there remains a significant gap in high-quality, large-scale research. Notably, while we found only a small number of RCTs addressing AI in cardiovascular care, and even fewer focused on hard clinical outcomes, the number of FDA-cleared AI-enabled cardiology applications already exceeds a hundred.^54^ This discrepancy reflects a broader challenge in the regulatory landscape, where clearance is often based on technical performance or retrospective validation without prospective data. Although a systematic assessment of FDA-cleared tools was beyond the scope of this review, future efforts should prioritize integrating robust clinical trial evidence into the approval pathway to ensure safety, efficacy, and clinical value. Given cardiology's strong reliance on evidence-based medicine, this lack of robust data is a key limitation, highlighting the need for more RCTs to assess AI's clinical impact. This also indicates that the implementation of AI in this field has been relatively slow. This is partly due to the limited AI-oriented management and education among cardiologists. Many clinicians lack the necessary training to incorporate AI into their practice effectively. Educational programs focused on AI could empower cardiologists to lead the integration of these tools, accelerating their adoption in routine care. Additionally, the heterogeneity observed in study outcome measures, patient populations, and AI applications complicates the ability to draw definitive conclusions across the literature. This variability not only poses challenges for synthesizing results but also limits the feasibility of conducting a meta-analysis and therefore standard publication bias assessments such as funnel plots or Egger’s test could not be applied. Lastly, given the rapid pace of AI research, development, and adoption, new studies may have been published after our systematic review search was completed.

## Conclusions

This review highlights AI's potential to enhance cardiovascular care through improved early detection, diagnostic accuracy, and resource efficiency. However, the limited number of RCTs indicates a need for more high-quality studies to validate AI’s effectiveness across various clinical domains.

Perspectives **COMPETENCY IN MEDICAL KNOWLEDGE AND PATIENT CARE:** This review highlights the potential of AI to improve clinical outcomes and operational efficiency in cardiology. By leveraging AI-guided analyses in modalities like ECG, echocardiography, and cardiac CT, clinicians can achieve earlier and more accurate diagnoses, tailor patient management strategies, and potentially reduce hospitalizations and adverse events.

**TRANSLATIONAL OUTLOOK:** To move from promise to practice, further large-scale, well-designed RCTs are essential to validate AI’s clinical benefits, ensure data quality, and address ethical and privacy considerations. Greater clinician education, standardization of outcome measures, and proactive involvement of hospitals and clinical leaders can foster sustainable integration of AI into routine cardiovascular care, ultimately enhancing patient trust and long-term outcomes.

### Declaration of Generative AI and AI-Assisted Technologies in the Writing Process

During the preparation of this work, the authors used ChatGPT for proofreading. After using this tool, the authors reviewed and edited the content as needed and take full responsibility for the publication's content.

## Funding support and author disclosures

The authors have reported that they have no relationships relevant to the contents of this paper to disclose.
