# Supplementary material for: Randomized Controlled Trials Evaluating Artificial Intelligence in Cardiovascular Care: A Systematic Review
Source: JACC Adv. 2025 Sep 24;4(11):102152. doi: 10.1016/j.jacadv.2025.102152 (PMC12506480; doi:10.1016/j.jacadv.2025.102152)
Supplement: Supplemental_Material [file mmc1.docx]

Supplemental Appendix 1 –Search strategy

**Medline Search Strategy (Inception - 29/11/2024):**

- **Search Terms:** (("artificial intelligence") OR ("deep learning") OR ("machine learning") OR ("neural networks")) OR (“Natural language processing “)) AND ((“Cardiology”) OR (“cardiovascular disease”) OR (“Heart failure”) OR (“Arrhythmia”) OR (“Echocardiography”) OR (“ECG”) OR (“Myocardial infarction”) OR (“Angiography”) OR (“Electrophysiology”) OR (“Cardiomyopathy”) OR (“Atrial fibrillation”) OR (“Heart transplant”) OR (“Pacemaker”) OR (“CRT”) OR (“coronary artery disease”)) AND ("randomized controlled trial") in MeSH terms and Title/Abstract
- **Filters Applied:** English language
- **Date of Search:** 29/11/2024.

**Web of Science Search Strategy (Inception - 29/11/2024):**

- **Search Terms:** (ALL=(“Artificial intelligence”)) OR ALL=(Machine learning)) OR ALL=(Deep learning)) OR ALL=(Neural networks)) OR ALL=(Natural language processing) AND (ALL=(“Cardiology” OR “Cardiovascular disease” OR “Heart failure” OR “Arrhythmia” OR “Echocardiography” OR “ECG” OR “Myocardial infarction” OR “Angiography” OR “Electrophysiology” OR “Cardiomyopathy” OR “Atrial fibrillation” OR “Heart transplant” OR “Pacemaker” OR “CRT” OR “Coronary artery disease”) AND (ALL=(Randomized controlled trial))
- **Filters Applied:** English language
- **Date of Search:** 29/11/2024.

**Cochrane Library Search Strategy (Inception - 29/11/2024):**

- **Search Terms:** (“Artificial intelligence” OR “Machine learning” OR “Deep learning” “Neural networks” OR “Natural language processing”) AND (“Cardiology” OR “cardiovascular disease” OR “Heart failure” OR “Arrhythmia” OR “Echocardiography” OR “ECG” OR “Myocardial infarction” OR “Angiography” OR “Electrophysiology” OR “Cardiomyopathy” OR “Atrial fibrillation” OR “Heart transplant” OR “Pacemaker” OR “CRT” OR “coronary artery disease”) AND ("randomized controlled trial")
- **Filters Applied:** English language
- **Date of Search:** 29/11/2024.

**Supplemental Appendix 2 - PRISMA 2020 Checklist**

| **Section and Topic** | **Item #** | **Checklist item** | **Location where item is reported** |
| --- | --- | --- | --- |
| **TITLE** | | |  |
| Title | 1 | Identify the report as a systematic review. | Page 1 |
| **ABSTRACT** | | |  |
| Abstract | 2 | See the PRISMA 2020 for Abstracts checklist. | Page 2 |
| **INTRODUCTION** | | |  |
| Rationale | 3 | Describe the rationale for the review in the context of existing knowledge. | Page 4 |
| Objectives | 4 | Provide an explicit statement of the objective(s) or question(s) the review addresses. | Page 4 |
| **METHODS** | | |  |
| Eligibility criteria | 5 | Specify the inclusion and exclusion criteria for the review and how studies were grouped for the syntheses. | Page 5 |
| Information sources | 6 | Specify all databases, registers, websites, organisations, reference lists and other sources searched or consulted to identify studies. Specify the date when each source was last searched or consulted. | Page 5 |
| Search strategy | 7 | Present the full search strategies for all databases, registers and websites, including any filters and limits used. | Page 5, supplement 1 |
| Selection process | 8 | Specify the methods used to decide whether a study met the inclusion criteria of the review, including how many reviewers screened each record and each report retrieved, whether they worked independently, and if applicable, details of automation tools used in the process. | Page 5 |
| Data collection process | 9 | Specify the methods used to collect data from reports, including how many reviewers collected data from each report, whether they worked independently, any processes for obtaining or confirming data from study investigators, and if applicable, details of automation tools used in the process. | Page 6 |
| Data items | 10a | List and define all outcomes for which data were sought. Specify whether all results that were compatible with each outcome domain in each study were sought (e.g. for all measures, time points, analyses), and if not, the methods used to decide which results to collect. | Page 6 |
|  | 10b | List and define all other variables for which data were sought (e.g. participant and intervention characteristics, funding sources). Describe any assumptions made about any missing or unclear information. | Page 18-24 |
| Study risk of bias assessment | 11 | Specify the methods used to assess risk of bias in the included studies, including details of the tool(s) used, how many reviewers assessed each study and whether they worked independently, and if applicable, details of automation tools used in the process. | Page 6 |
| Effect measures | 12 | Specify for each outcome the effect measure(s) (e.g. risk ratio, mean difference) used in the synthesis or presentation of results. | N/A |
| Synthesis methods | 13a | Describe the processes used to decide which studies were eligible for each synthesis (e.g. tabulating the study intervention characteristics and comparing against the planned groups for each synthesis (item #5)). | NA |
|  | 13b | Describe any methods required to prepare the data for presentation or synthesis, such as handling of missing summary statistics, or data conversions. | NA |
|  | 13c | Describe any methods used to tabulate or visually display results of individual studies and syntheses. | NA |
|  | 13d | Describe any methods used to synthesize results and provide a rationale for the choice(s). If meta-analysis was performed, describe the model(s), method(s) to identify the presence and extent of statistical heterogeneity, and software package(s) used. | NA |
|  | 13e | Describe any methods used to explore possible causes of heterogeneity among study results (e.g. subgroup analysis, meta-regression). | NA |
|  | 13f | Describe any sensitivity analyses conducted to assess robustness of the synthesized results. | NA |
| Reporting bias assessment | 14 | Describe any methods used to assess risk of bias due to missing results in a synthesis (arising from reporting biases). | Page 6 |
| Certainty assessment | 15 | Describe any methods used to assess certainty (or confidence) in the body of evidence for an outcome. | NA |


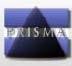
 **PRISMA 2020 Checklist**

| **Section and Topic** | **Item #** | **Checklist item** | **Location where item is reported** |
| --- | --- | --- | --- |
| **RESULTS** | |  |  |
| Study selection | 16a | Describe the results of the search and selection process, from the number of records identified in the search to the number of studies included in the review, ideally using a flow diagram. | Page 7-12 |
|  | 16b | Cite studies that might appear to meet the inclusion criteria, but which were excluded, and explain why they were excluded. | NA |
| Study characteristics | 17 | Cite each included study and present its characteristics. | Page 7-12 |
| Risk of bias in studies | 18 | Present assessments of risk of bias for each included study. | Page 11-12 |
| Results of individual studies | 19 | For all outcomes, present, for each study: (a) summary statistics for each group (where appropriate) and (b) an effect estimate and its precision (e.g. confidence/credible interval), ideally using structured tables or plots. | Page 12-14 |
| Results of syntheses | 20a | For each synthesis, briefly summarise the characteristics and risk of bias among contributing studies. | Page 11-12 |
|  | 20b | Present results of all statistical syntheses conducted. If meta-analysis was done, present for each the summary estimate and its precision (e.g.  confidence/credible interval) and measures of statistical heterogeneity. If comparing groups, describe the direction of the effect. | NA |
|  | 20c | Present results of all investigations of possible causes of heterogeneity among study results. | N/A |
|  | 20d | Present results of all sensitivity analyses conducted to assess the robustness of the synthesized results. | N/A |
| Reporting biases | 21 | Present assessments of risk of bias due to missing results (arising from reporting biases) for each synthesis assessed. | Page 11-12 |
| Certainty of evidence | 22 | Present assessments of certainty (or confidence) in the body of evidence for each outcome assessed. | NA |
| **DISCUSSION** | |  |  |
| Discussion | 23a | Provide a general interpretation of the results in the context of other evidence. | Page 12 |
|  | 23b | Discuss any limitations of the evidence included in the review. | Page 15-16 |
|  | 23c | Discuss any limitations of the review processes used. | Page 15-16 |
|  | 23d | Discuss implications of the results for practice, policy, and future research. | Page 12-16 |
| **OTHER INFORMATION** | |  |  |
| Registration and protocol | 24a | Provide registration information for the review, including register name and registration number, or state that the review was not registered. | Page 5 |
|  | 24b | Indicate where the review protocol can be accessed, or state that a protocol was not prepared. | Page 5 |
|  | 24c | Describe and explain any amendments to information provided at registration or in the protocol. | Page 5 |
| Support | 25 | Describe sources of financial or non-financial support for the review, and the role of the funders or sponsors in the review. | Page 1 |
| Competing  interests | 26 | Declare any competing interests of review authors. | Page 1 |
| Availability of data, code and other materials | 27 | Report which of the following are publicly available and where they can be found: template data collection forms; data extracted from included studies; data used for all analyses; analytic code; any other materials used in the review. | NA |

*From:*  Page MJ, McKenzie JE, Bossuyt PM, Boutron I, Hoffmann TC, Mulrow CD, et al. The PRISMA 2020 statement: an updated guideline for reporting systematic reviews. BMJ 2021;372:n71. doi:

10.1136/bmj.n71

# Supplemental Table 3. Summary of Randomization Methods in Included Trials

| Study Title | Randomization Method | Notes |
| --- | --- | --- |
| Lin et al (2024a) | Simple randomization based on hospital record numbers | Randomized before trial; ensured patient-level continuity |
| Yao et al | Cluster randomization stratified by region and volume | Conducted at care team level |
| He et al | 1:1 non-block randomization | Performed once at trial start |
| Hill et al | Simple 1:1 randomization, centrally by statistician | Individual-level |
| De Backer et al | 1:1 stratified per site | Site-based stratification |
| Sandhu et al | Permuted block randomization | 1:1 allocation |
| Yang et al | Fixed block randomization via computerized system | Independent allocation |
| Huang et al | Consecutive randomization using number table | Patient-level |
| Adedinsewo et al | Dynamic minimization stratified by site via web platform | Real-time allocation |
| Lin et al (2024b) | Simple 1:1 based on ECG date (odd/even) | Independent sequence pre-generated |
| Upton et al | Random block stratified by sex, center, and CAD status | Allocation by independent data manager |

| Author | Protocol Registered (ClinicalTrials.gov ) | Code Availability | Data Availability | Declaration of adherence to reporting framework guidelines | Name of guideline (SPIRIT-AI/e CONSORT-AI...) |
| --- | --- | --- | --- | --- | --- |
| Lin et al (2024a) | yes, ClinicalTrials.gov NCT05118035 | yes | no | yes | CONSORT-AI |
| Yao et al | yes, ClinicalTrials.gov  NCT04000087 | no | no | yes | CONSORT-AI |
| He et al | Yes, ClinicalTrials.gov NCT05140642 | Yes | Yes | Yes | CONSORT-AI and SPIRIT-AI |
| Hill et al | Yes, ClinicalTrials.gov NCT04045639 | No | Yes | no |  |
| De Backer et al | Yes, ClinicalTrials.gov NCT04180605 | no | no | no |  |
| Sandhu et al | Yes, ClinicalTrials.gov NCT04789278 | no | Yes | no |  |
| Yang et al | Yes, ClinicalTrials.gov NCT03901326 | no | Yes | no |  |
| Huang et al | No | no | no | no |  |
| Adedinsewo et al | Yes, ClinicalTrials.gov NCT05438576 | yes | yes | yes | CONSORT-AI |
| Lin et al (2024b) | Yes, ClinicalTrials.gov NCT05118009 | no | no | Yes | CONSORT-AI |
| Upton et al | Yes, ClinicalTrials.gov  NCT05028179 | no | no | no |  |

# Supplemental Table 4. Summary of adherence to AI-related guidelines in Included Trials

# Supplemental Table 5. Reporting Guidelines Relevant to AI-Based Clinical Trials in Cardiovascular Care

| **Guideline** | **Focus Area** | **Use in AI-RCTs** | **Source** |
| --- | --- | --- | --- |
| CONSORT-AI | Reporting AI RCT results | Primary tool for reporting structure | <https://doi.org/10.1038/s41591-020-1034-x> |
| SPIRIT-AI | Trial protocol design | For assessing protocol quality and planning | <https://doi.org/10.1038/s41591-020-1037-7> |
| TRIPOD-AI / TRIPOD-ML | Prediction model transparency | For evaluating prediction model use within RCT | https://www.tripod-statement.org |
| STARD-AI | Diagnostic test performance | For trials assessing diagnostic AI | https://www.birmingham.ac.uk/research/centre-for-patient-reported-outcomes/research/stard-ai.aspx |
| PROBAST-AI | Risk of bias in prediction models | For evaluating robustness and validity of AI models | https://www.probast.org |
| DECIDE-AI | Feasibility/pilot testing of AI | For assessing pre-RCT pilot trials | https://doi.org/10.1038/s41591-022-01699-z |
| CLAIM | Radiology/medical imaging AI | If the AI tool uses imaging data | https://doi.org/10.1148/ryai.2020200029 |
| PRIME | Cardiovascular imaging | For AI trials involving cardiac imaging (CT, Echo) | https://doi.org/10.1016/j.jcmg.2022.10.015 |
|  |  |  |  |
|  |  |  |  |
| This table summarizes established and emerging reporting frameworks designed to support the development, evaluation, and transparent reporting of clinical trials involving artificial intelligence (AI) tools. While most of these guidelines—such as CONSORT-AI and SPIRIT-AI—are domain-agnostic and applicable across medical specialties, PRIME is specifically tailored to the evaluation of AI tools in cardiovascular imaging. Other frameworks, such as CLAIM (radiology-focused), TRIPOD-ML, and STARD-AI (diagnostic performance), provide valuable structure for assessing methodological rigor in trials that utilize imaging, prediction, or diagnostic AI tools commonly applied in cardiology. Together, these resources offer a comprehensive reference to improve transparency, reproducibility, and clinical relevance in AI-based randomized controlled trials, including those in cardiovascular care. | | | |
